# Metagenomic data of the microbial community of lab-scale nitritation-anammox sequencing-batch bioreactor performing nitrogen removal from synthetic wastewater

**DOI:** 10.1016/j.dib.2019.104722

**Published:** 2019-11-04

**Authors:** Andrey V. Mardanov, Roman V. Kotlyarov, Alexey V. Beletsky, Yury A. Nikolaev, Anna Yu. Kallistova, Vladimir A. Grachev, Yulia Yu Berestovskaya, Nikolai V. Pimenov, Nikolai V. Ravin

**Affiliations:** a Institute of Bioengineering, Research Center of Biotechnology of the Russian Academy of Sciences, Moscow, Russia; b Winogradsky Institute of Microbiology, Research Center of Biotechnology of the Russian Academy of Sciences, Moscow, Russia

**Keywords:** Anammox, Metagenome, *Candidatus* brocadia

## Abstract

The nitritation-anammox process, which involves partial aerobic oxidation of the ammonium to nitrite and following oxidation of ammonium by nitrite to molecular nitrogen, is an efficient and cost-effective approach for biological nitrogen removal from wastewater. To characterize the microbial communities involved in the nitrogen and carbon cycles in wastewater treatment bioreactors employing this process, we sequenced the metagenome of a sludge sample collected from the lab-scale nitritation-anammox sequencing-batch reactor. At the phylum level, Proteobacteria and Chloroflexi were the most numerous groups. Anammox bacteria belonged to the genus *Candidatus* Brocadia. The obtained data will help to investigate the taxonomical and functional diversity the microbial communities involved in nitritation-anammox process, and will be used for genome-based analysis of uncultured bacterial lineages. The raw sequencing data is available from the NCBI Sequence Read Archive (SRR9831403) database under the BioProject PRJN0A55627.

**Table:** Specifications Table

Subject area	Biology
More specific subject area	Metagenomics
Type of data	Metagenomic sequences
How data was acquired	Shotgun DNA sequencing using Illumina HiSeq 2500
Data format	Raw and analyzed
Experimental factors	Microbiological and technological features of the lab-scale nitritation-anammox sequencing-batch bioreactor
Experimental features	The sludge sample from the laboratory bioreactor treating artificial wastewater from nitrogen was collected and then the total community DNA was extracted and sequenced.
Data source location	Winogradsky Institute of Microbiology, Research Center of Biotechnology of the Russian Academy of Sciences, Moscow, Russia, Date of sample collection - January 28, 2019
Data accessibility	Data is submitted to NCBI with BioProject PRJN0A55627 and it is in the public repository. The direct URL to data is https://www.ncbi.nlm.nih.gov/sra?linkname=bioproject_sra_all&from_uid=556270

**Table:** 

**Value of the Data** • The obtained data provide insight into the composition of the microbial community involved in nitritation-anammox process. • This metagenome is valuable for the study of microbial processes in wastewater treatment bioreactors. • The obtained data could be used for genome-based analysis of uncultured anammox bacteria. • The metagenome data is available for further analysis and comparison of metagenomes among various wastewater treatment plants.

## 1 Data

Technologies of biological nitrogen removal from wastewater based on the nitritation-anammox process, which involves partial aerobic oxidation of the ammonium to nitrite (nitritation) and anaerobic oxidation of ammonium by nitrite producing molecular nitrogen (anammox), has been actively developed in recent decades [1]. The key role in this process is played by the anammox bacteria, a distinct lineage of the phylum Planctomycetes [2]. Metagenomic studies revealed other members of anammox microbial communities, which usually include microorganisms performing nitrification and denitrification, as well as various heterotrophs [1,3].

**Fig. 1:**
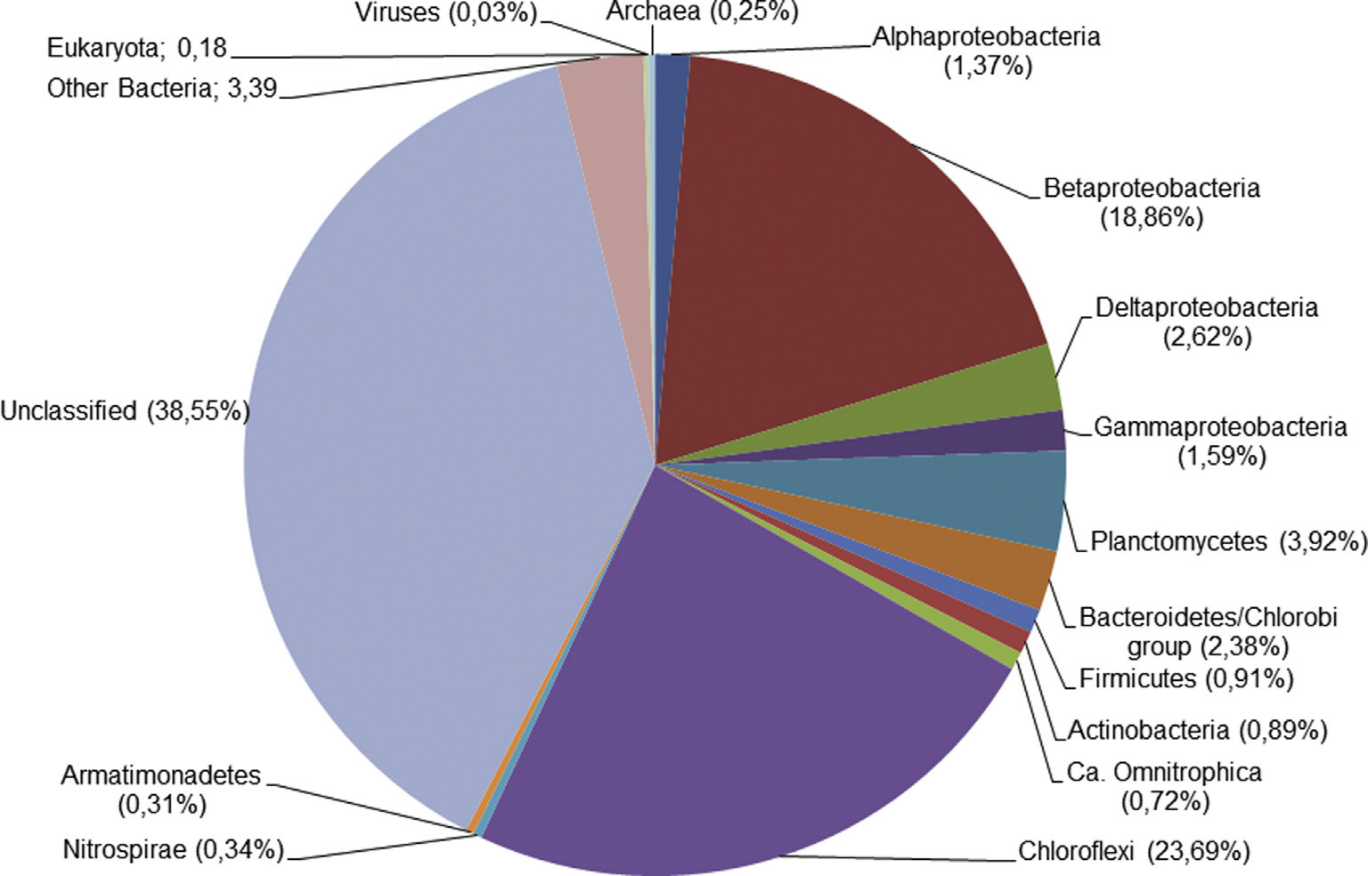
Taxonomic assignment of the metagenome of activated sludge of the nitritation-anammox bioreactor.

We sequenced the metagenome of a sludge sample collected from the nitritation-anammox sequencing-batch reactor. Data on the taxonomic assignment of the metagenome are shown in Fig. 1. Of the obtained 15, 910, 349 sequencing reads, 60.97% were assigned to Bacteria, 0.25% to Archaea, 0.18% to Eukaryota, 0.03% to viruses, while other reads were not classified. At the phylum level, *Proteobacteria* (24.43%, mostly members of the class beta, 18.86%), *Chloroflexi* (23.69%) and *Planctomycetes* (3.92%) were the most abundant. as shown in Fig. 1. At the species level the most abundant was betaproteobacterium *Thauera phenylacetica*, accounted for 9.78% of all reads. This bacterium can degrade aromatic compounds under denitrifying conditions [6]. Among the *Chloroflexi*, bacteria closely related to uncultured Chloroflexi bacterium OLB14 [3], Anaerolineae bacterium UTCFX1 and Anaerolineae bacterium UTCFX3 accounted for 8.09%, 2.73% and 2.66% of sequencing reads, respectively. These genomes were assembled from the sludge metagenomes of wastewater treatment plants. About 3.48% of sequencing reads were assigned to the anammox bacteria of the genus *Candidatus* Brocadia. The obtained data will help investigate the diversity and ecology of the microbial community in the wastewater treatment bioreactor and could be used for genome-based analysis of uncultured microbial lineages, including anammox bacteria.

## 2 Experimental design, materials, and methods for metagenome investigation

### 2.1 Sample collection and preparation

The object of this study was a microbial community of activated sludge developed in a sequencing batch nitritation-anammox bioreactor with a volume of 9 L. A working cycle of 6 hours consisted of a sedimentation stage (20 min), followed by the supply the artificial wastewater solution (30 min), and aeration/mixing stage (5 h 10 min). Discharge of treated water took place simultaneously with fresh feeding solution income. During the operation cycle, 2 L of artificial wastewater solution [4] containing 200 mg NH4–N per L (pH 8.3) was supplied. Non-woven polyethylene fibre carrier was used for retention of the biomass. The reactor was kept at a temperature of 30 °C and an oxygen concentration of 0.4–0.6 mg/L. Activated sludge from the previously described anammox reactor served as an inoculum [5]. The process of cultivation of activated anammox sludge was carried out for 5 months. During this period, the nitrogen removal efficiency increased from 30% to 90%. The sample of firmly attached sludge (50 g) was collected on day 150 of the start of reactor operation when it stably removed about 90% of the total nitrogen with an influent ammonium concentration of 200 mg NH4–N per liter.

### 2.2 DNA extraction

Metagenomic DNA was isolated from the fresh sludge sample using Power Soil DNA Isolation Kit (MO BIO Laboratories Inc, Carlsbad, USA). The quality and concentration of the extracted DNA sample was measured using Qubit® dsDNA HS Assay Kit (Life Technologies), followed by agarose gel electrophoresis. About 6 μg of total DNA (83.7 ng/μL) was isolated.

### 2.3 Sequencing and taxonomic analysis

100 ng of DNA was used to prepare the sequencing library using TruSeq nano DNA Library prep Kit (Illumina Inc., USA) following the manufacturer's instructions. The sequencing of this library on the Illumina HiSeq-2500 instrument using HiSeq Rapid SBS Run v2 sequencing reagents generated 49,999,055 read pairs (2 × 150 nt, about 15 Gbp in total). Primer removal and quality trimming were performed with Cutadapt v. 1.17 [7] and Sickle v. 1.33 (https://github.com/najoshi/sickle), respectively. Cutadapt was used with the default settings, and Q30 score was used for the Sickle. Overlapping paired-end reads were merged using FLASH [8]. The final dataset consisted of 79,557,279 sequences (11,528,532,853 bases), of them 15,910,349 represented merged reads (3,009,936,782 bases).

Taxonomic classification of metagenomic sequences was carried out using the Kaiju program [9], with default parameters. In order to improve the efficiency of taxonomic classification only longer sequences obtained from merged reads were used. A microbial subset of the NCBI non-redundant protein database, also including fungi and microbial eukaryotes (nr + euk option), was used as a reference database. 9,780,729 of 15,910,349 sequences were classified.

## Conflict of interest

The authors declare that they have no known competing financial interests or personal relationships that could have appeared to influence the work reported in this paper.
